# Supplementary material for: Cost-effectiveness of a “treat-all” strategy using Direct-Acting Antivirals (DAAs) for Japanese patients with chronic hepatitis C genotype 1 at different fibrosis stages
Source: PLoS One. 2021 Apr 1;16(4):e0248748. doi: 10.1371/journal.pone.0248748 (PMC8016275; doi:10.1371/journal.pone.0248748)
Supplement: S1 Materials — (DOCX) [file pone.0248748.s001.docx]

**S1 Materials**

Structure of the Markov model and stage-specific fibrosis progression rates in the natural history model of chronic hepatitis C virus infection

Our natural history model of chronic hepatitis by HCV consisted of 4 fibrosis stages in patient with hepatitis—i.e., F0, F1, F2, F3, and F4 (where F4 corresponds to liver cirrhosis)—and these were combined with decompensated cirrhosis, hepatocellular carcinoma, and death state.

To determine the stage-specific fibrosis progression rates in the Markov model, we applied the equations developed by the meta-regression model in the meta-analysis by Thein HH et. al. (1). The equations are shown in S1 Table.

**S1 Table Meta-regression algorithm**

exp: exponential function; HCV: hepatitis C virus; IDU: injection drug use.

To calibrate parameters of the equations, we constructed a Markov model of the natural history on TreeAge Pro (R) and applied the findings for a cohort of patients with chronic hepatitis of stages F0, F1, F2, and F3 to the model to simulate their prognosis. We used the cumulative incidence of liver cirrhosis to adjust the parameters of the equations according to the simulation by the model. The most influential parameter was the duration of the HCV infection followed by the proportion of patients infected by blood transfusion, the proportion of patients with genotype one, ranked by the magnitude of change in the estimated incidence, and we determined their value in order within the range that could be assumed from the results of Japanese epidemiological studies (2).

The values of parameters of the algorithm which most fit the cumulative incidence curve were as follows: the duration of infection, the proportions of HCV patients with genotype one, with infection due to blood transfusion, with a drug habit involving injection, or with heavy alcohol use were 27.5 years, 0.8, 0.395, 0.05, 0.181, respectively. The proportions of patients with blood transfusion (BTF) and injection drug users (IDU) were compatible with the results of an epidemiological study in which the BTF and IDU proportions were 0.35 and 0.06, respectively (3).

The corresponding transition rates of fibrosis stages were as follows: those from F0 to F1, F1 to F2, F2 to F3, F3 to F4 were 0.034, 0.046, 0.062, 0.059, respectively.

S1 Figure shows the estimated incidence of liver cirrhosis from the model and the actual incidence.

To assess the validity of our Markov model with transition probabilities of fibrosis stage derived by the multivariate algorithm expecting the natural history of the patients with HCV infection, we compared the cumulative survival rates of another cohort of the patients with HCV hepatitis (4). When we applied those fibrosis rates to the natural history model to predict the survival rates at 5 years and 10 years, the predicted and actual survival rates were 0.93 and 0.95 at 5 years and 0.80 and 0.77 at 10 years, respectively, and the life years predicted by the model and calculated from the original survival curve by 12 years were 10.8 and 10.7, respectively. Therefore, we considered that the fibrosis rates obtained from Ikeda’s study were appropriate for the natural history model.

**S1 Figure The estimated incidence of liver cirrhosis**


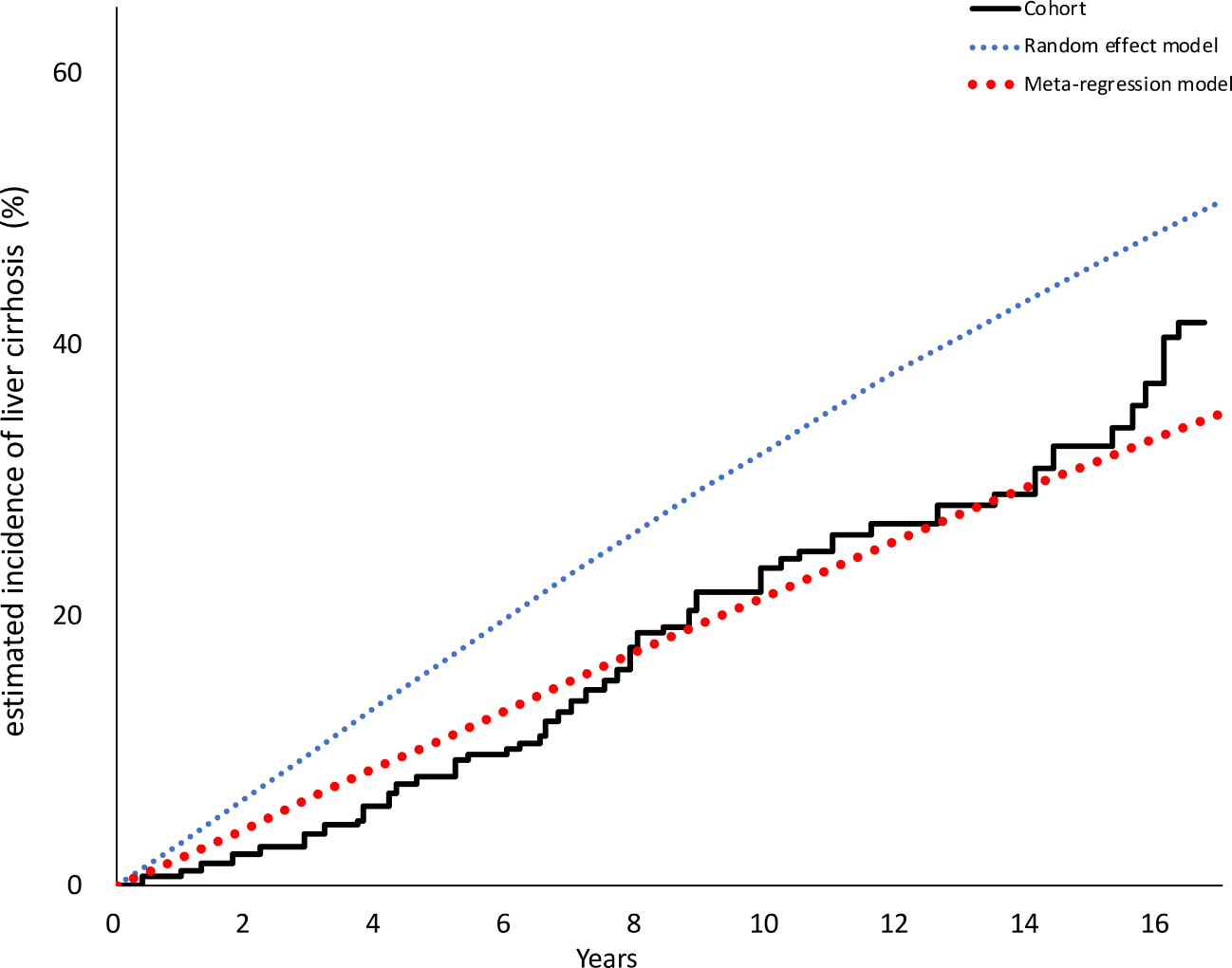


**References**

1. Thein HH, Yi Q, Dore GJ, Krahn MD. Estimation of stage-specific fibrosis progression rates in chronic hepatitis C virus infection: a meta-analysis and meta-regression. Hepatology. 2008;48(2):418–31.

2. Ikeda K, Saitoh S, Suzuki Y, Kobayashi M, Tsubota A, Koida I, et al. Disease progression and hepatocellular carcinogenesis in patients with chronic viral hepatitis: a prospective observation of 2215 patients. Journal of hepatology. 1998;28(6):930–8.

3. Liakina V, Hamid S, Tanaka J, Olafsson S, Sharara AI, Alavian SM, et al. Historical epidemiology of hepatitis C virus (HCV) in select countries - volume 3. Journal of viral hepatitis. 2015;22 Suppl 4:4–20.

4. Kasahara A, Tanaka H, Okanoue T, Imai Y, Tsubouchi H, Yoshioka K, et al. Interferon treatment improves survival in chronic hepatitis C patients showing biochemical as well as virological responses by preventing liver-related death. Journal of viral hepatitis. 2004;11(2):148–56.
